# Contralateral knee osteoarthritis severity relates to magnetic resonance imaging findings in knees with and without osteoarthritis: Data from the osteoarthritis initiative

**DOI:** 10.1016/j.ocarto.2025.100585

**Published:** 2025-02-17

**Authors:** Jeffrey B. Driban, Jonggyu Baek, Julieann C. Patarini, Emily Kirillov, Nhung Vo, Michael J. Richard, Ming Zhang, Matthew S. Harkey, Grace H. Lo, Shao-Hsien Liu, Charles B. Eaton, Jamie MacKay, Mary F. Barbe, Timothy E. McAlindon

**Affiliations:** a Department of Population and Quantitative Sciences, UMass Chan Medical School, 55 Lake Avenue North, Worcester, MA, 01655, USA; b Division of Rheumatology, Allergy, & Immunology, Tufts Medical Center, 800 Washington, Street Boston, MA, 02111, USA; c Department of Computer Science, Boston University, 111 Cummington Mall, Boston, MA, 02215, USA; d Department of Kinesiology, Michigan State University, 308 W. Circle Drive, East Lansing, MI, 48824, USA; e Department of Medicine, Baylor College of Medicine, One Baylor Plaza, Houston, TX, 77030, USA; f Medical Care Line and Research Care Line, Houston VA HSR&D Center for Innovations in Quality, Effectiveness and Safety, Michael E. DeBakey Medical Center, 2002 Holcombe Blvd, Houston, TX, 77030, USA; g Center for Primary Care and Prevention, Kent Hospital, 111 Brewster St, Pawtucket, RI, 02860, USA; h Brown University School of Public Health, 121 South Main Street Providence, RI, 02903, USA; i Department of Radiology, University of Cambridge, Box 218, Cambridge Biomedical Campus, Cambridge, CB2 0QQ, United Kingdom; j Norwich Medical School, University of East Anglia, Norwich Research Park, Norwich, NR4 7TJ, United Kingdom; k Aging + Cardiovascular Discovery Center, Lewis Katz School of Medicine, Temple University, 3500 N. Broad Street, Philadelphia, PA, 19140, USA; l Division of Rheumatology, Department of Medicine, UMass Chan Medical School, 55 Lake Avenue North, Worcester, MA, 01655, USA

**Keywords:** Magnetic resonance imaging, Osteoarthritis, Knee

## Abstract

**Objective:**

We explored whether a magnetic resonance imaging (MRI)-based composite score of bone marrow lesion and effusion-synovitis volumes related to contralateral knee osteoarthritis disease severity.

**Design:**

Using data from the Osteoarthritis Initiative, we conducted cross-sectional knee-based analyses among participants with bilateral knee MRIs and at least one knee with Kellgren–Lawrence (KL) grade ≥1 and a WOMAC pain score ≥10/100 (n ​= ​693). Bone marrow lesion and effusion-synovitis volumes on MRIs were used to calculate a composite score (“disease activity”). We divided the disease activity score into tertiles. We used multinomial logistic models to explore the association between disease activity in knees with and without radiographic osteoarthritis (outcome) and the contralateral disease severity (KL grade or disease activity; exposure).

**Results:**

We included 1386 knees from participants with an average age of 62 (standard deviation ​= ​9) years. Most participants were overweight and had mild-to-moderate radiographic osteoarthritis. Disease activity among knees without radiographic osteoarthritis had statistically significant relationships with contralateral disease activity (range of odds ratios: 4.86–23.22) but not contralateral KL grade (range of odds ratios: 0.86–1.01). Disease activity among knees with radiographic osteoarthritis had statistically significant relationships with contralateral disease activity and KL grade; however, the association was stronger for contralateral disease activity than KL grade (range of odds ratios: 3.67–21.29 versus 1.96–2.20).

**Conclusion:**

Structural findings in one knee may relate to structural findings in the other knee. This highlights the need for future studies to explore how the contralateral knee could impact clinical trial screening, monitoring, and intervention strategies, especially when testing localized therapies.

## 1 Introduction

Knee osteoarthritis is a leading cause of pain and disability with few effective treatments, which mainly offer small-moderate treatment effects and significant adverse effects [1]. Because we lack effective nonsurgical treatments for knee osteoarthritis pain, >50 ​% of people with knee osteoarthritis will undergo a total knee arthroplasty [2], with 40–50 ​% of those individuals having a contralateral knee arthroplasty within ten years [3,4], costing ∼$12 billion/year [5]. Given the urgent needs of people with knee osteoarthritis, it is critical to understand why we fail to achieve optimal treatment outcomes, especially with elusive disease-modifying therapies.

A significant impediment to our current treatment strategies and clinical trials for people with knee osteoarthritis is focusing only on one knee, often ignoring the contralateral knee. Failing to address the contralateral knee may explain why many localized therapeutic approaches fail to achieve optimal results. Pre-planned analyses of a clinical trial of an intra-articular injection of a Wnt-pathway inhibitor revealed that participants with unilateral knee symptoms experienced greater improvements in medial joint space width and self-reported pain and function than the placebo cohort, despite the overall study results being null [6]. In a follow-up trial, the investigators only recruited people with unilateral symptomatic knee osteoarthritis and detected a significant effect of the intra-articular treatment on self-reported knee pain and function [7].

The contralateral knee is unlikely an innocent bystander. In a clinical trial, 91 ​% of participants with symptomatic moderate-severe radiographic knee osteoarthritis had an equal or worse radiographic severity grade in the less painful knee [6]. Radiographically normal knees with contralateral radiographic knee osteoarthritis have a higher prevalence of Hoffa synovitis (69 ​% vs. 46 ​%), bone marrow lesions (BMLs; 76 ​% vs. 41–61 ​%), and cartilage lesions (98 ​% vs. 76–78 ​%) than knees with bilateral radiographically normal knees [8]. While there is a well-documented relationship, it remains unclear if relying on radiographs to assess the disease severity of the contralateral knee is sufficient because radiographs may lack the sensitivity to provide a robust assessment of the disease state [9]. A more fruitful screening tool for the contralateral knee disease state is likely magnetic resonance (MR) imaging, especially MR-based measures of dynamic disease processes (i.e., BMLs and effusion-synovitis) that are present during early- and late-stage knee osteoarthritis [10] and predict progression (e.g., cartilage loss) [10,11]. We recently developed and validated a composite MR-based score of BML and effusion-synovitis volumes, which relates to knee pain and may be a valuable tool for assessing OA in both knees (hereafter termed “disease activity”) [12].

We explored whether an MR-based composite score of bone marrow lesion and effusion-synovitis volumes relates to the severity of contralateral knee osteoarthritis when assessed with radiographic severity grades or MR-based measures. We hypothesized that an MR-based disease activity score relates to contralateral disease severity based on radiographic assessments (Kellgren–Lawrence grade) and the new MR-based disease activity score. MR-based biomarkers are more sensitive than radiographs, and hence, we hypothesized that the relationship would be stronger when assessing the contralateral knee with MR-based disease activity than when assessing a radiographic assessment. We explored these relationships among knees with and without radiographic osteoarthritis to understand if contralateral knee screening may be beneficial in future trials to modify osteoarthritis progression versus prevent osteoarthritis, respectively. Understanding the relationship between structural findings in a knee and the contralateral knee could inform future innovative clinical trials for knee osteoarthritis disease modification that consider the importance of screening, monitoring, and intervening on both knees to optimize outcomes.

## 2 Materials and methods

### 2.1 Study design

**Fig. 1:**
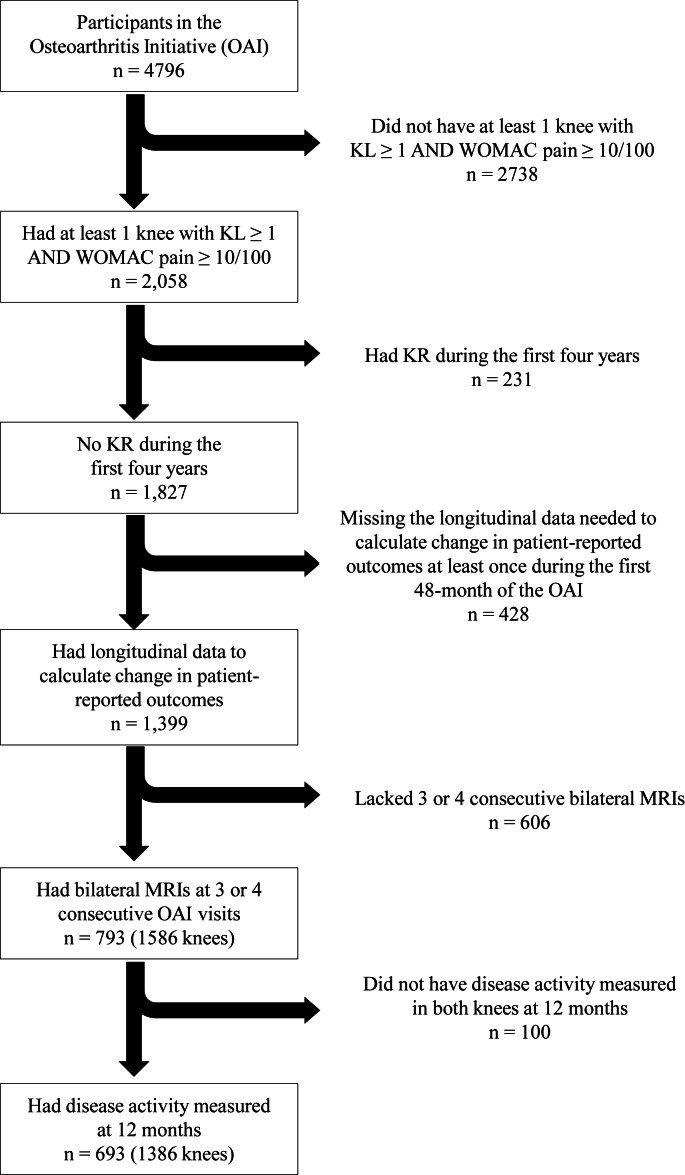
**Flow diagram depicting how participants from the Osteoarthritis Initiative were selected for the current analyses.** Abbreviations: KL ​= ​Kellgren–Lawrence grade, MRI ​= ​magnetic resonance imaging.

We conducted cross-sectional knee-based analyses of a subset of knees within a nested cohort study of participants we included from the Osteoarthritis Initiative (OAI; Fig. 1). Each person contributed two observations: 1) right knee ​= ​study knee (outcome) and left knee ​= ​contralateral knee (exposure), and 2) right knee ​= ​contralateral knee (exposure) and left knee ​= ​study knee (outcome).

### 2.2 Osteoarthritis initiative

The OAI is a multicenter cohort study of people in the United States with or at risk for symptomatic knee osteoarthritis. Study staff recruited 4796 men and women (ages 45 to 79) between February 2004 and May 2006 at four clinical sites: 1) University of Maryland and Johns Hopkins University, 2) Memorial Hospital of Rhode Island, 3) The Ohio State University, and 4) the University of Pittsburgh). Institutional review boards approved the OAI study at each clinical site and the coordinating center: Memorial Hospital of Rhode Island Institutional Review Board, The Ohio State University's Biomedical Sciences Institutional Review Board, University of Pittsburgh Institutional Review Board, University of Maryland Baltimore – Institutional Review Board, and Committee on Human Research at University of California, San Francisco. Each participant provided informed consent.

### 2.3 Nested cohort

For our overall project, we included people who had a knee with 1) Kellgren–Lawrence grade ≥1; 2) a WOMAC pain score ≥10/100; 3) no knee replacement before the 48-month OAI visit; and 4) longitudinal data to assess changes in patient-reported outcomes and walking speed at least once during the observation period (12–48-month visits; Fig. 1). We omitted the first year of OAI to avoid people who may regress to the mean in knee symptoms. We required participants to have bilateral MR images at 3 or 4 consecutive OAI visits because the overall project explored how MR-based biomarkers in both knees related to a person-level outcome.

### 2.4 Subsample used in current analyses

For our current analyses, we excluded 100 participants without bilateral 12-month disease activity measurements. Our eligible study sample for these cross-sectional analyses of data from the 12-month OAI visit included 1386 knees (n ​= ​693 participants). The presented analyses were deemed not human research by the UMass Chan Medical School Institutional Review Board.

### 2.5 Knee radiographs

Participants completed bilateral weight-bearing, fixed-flexion posterior–anterior knee radiographs. Central readers for the OAI provided Kellgren–Lawrence grades (0–4). The Kellgren–Lawrence grades had good agreement between two readings separated by 3–9 months (kappa coefficients between 0.70 and 0.78; n ​= ​150). We relied on read project 15 in the OAI public files: kxr_sq_bu01 (versions 1.8). We defined radiographic knee osteoarthritis as a knee with Kellgren–Lawrence grade ≥2.

### 2.6 MR imaging acquisition

Participants underwent MR imaging at each OAI site using Siemens 3.0 ​T Trio MR systems and knee coils. Study-certified, licensed MR technicians regularly conducted quality control measurements to maintain quality standards and consistency among MR equipment.

Acquisitions included an intermediate-weighted fat-suppressed (IWFS) sequence (field of view ​= ​160 ​mm, slice thickness ​= ​3 ​mm, skip ​= ​0 ​mm, flip angle ​= ​180°, echo time ​= ​30 ​ms, recovery time ​= ​3200 ​ms, 313 ​× ​448 matrix, x-resolution ​= ​0.357 ​mm, y-resolution ​= ​0.511 ​mm), which was used to measure BML and effusion-synovitis volumes.

### 2.7 Measuring bone marrow lesions

We used our validated semi-automated software to measure BML volumes in six regions: the medial and lateral patella, tibia, and femur [13]. We had one reader focus on patellar BML measurements and another on tibiofemoral BML measurements. Each reader manually identified BMLs and adjusted the threshold to modify the computer-generated BML segmentation. An experienced third reader led weekly quality assurance meetings to suggest adjustments to ensure measurement consistency. Each reader had excellent intra-reader reliability (intra-class correlation coefficients [3,1 model] >0.95; n ​= ​20). Our primary BML outcomes were six regional BML volumes, each representing the sum of the BML volume within each anatomic region (medial and lateral: patella, distal femur, proximal tibia).

### 2.8 Measuring effusion-synovitis

One reader measured effusion-synovitis with our validated semi-automated software [14]. The reader used the software to manually adjust thresholds to segment regions of high-signal intensity and remove irrelevant areas of high-signal intensity (e.g., subchondral cysts, blood vessels). The reader met weekly with another experienced effusion-synovitis reader to ensure quality and consistency among measurements. The reader had excellent intra-reader reliability (intra-class correlation coefficient [3, 1 model] ​= ​0.97; n ​= ​20). Our primary effusion-synovitis volume represented effusion-synovitis throughout the entire knee.

### 2.9 Calculating disease activity score

We calculated disease activity based on our validated algorithm [12]. First, we adjusted each MR-based measure for a person's bone width (distance between medial and lateral femoral epicondyle). This adjustment allowed us to account for variations in knee size. Next, we standardized each measure by subtracting the mean from a reference sample and dividing the difference by the standard deviation from that reference sample. This step ensured that all measurements were on the same scale to enable the calculation of disease activity and enhance interpretability. Finally, we summed the standardized measures in effusion-synovitis and the BML volumes for the six regions (medial and lateral: distal femur, proximal tibia, and patella). Higher values indicate worse disease.

### 2.10 Participant characteristics

Participants reported their gender (male, female, refused), date of birth (to calculate age), and race (open-ended question). The study staff measured a participant's weight and height to calculate their body mass index (kg/m^2^). Frequent knee symptoms for each knee were defined by an affirmative answer to “During the last 12 months, have you had pain, aching, or stiffness in or around your right knee on most days for at least one month? By most days, we mean more than half the days of a month.” The same question was asked for the left knee.

### 2.11 Statistical analysis

For our primary analyses, we used four separate multinomial logistic regression models. The outcome for each model was disease activity divided into tertiles based on data from all knees (low [reference], moderate, high). The first two models were performed among knees without radiographic knee osteoarthritis and examined contralateral Kellgren–Lawrence grade (3 levels: 0 or 1 [reference], 2, and 3 or 4) or contralateral disease activity (3 levels: low, moderate, high) as the exposures. Each model was adjusted for gender, age, and study knee side. Similar models were performed among knees with radiographic knee osteoarthritis. To further understand these relationships, we performed exploratory analyses stratified by Kellgren–Lawrence grade (0, 1, 2, and 3 or 4) in the study (outcome) knee and history of knee injury. We also replicated the primary analyses with the key components of disease activity (effusion-synovitis and BML volumes) to determine if they have similar associations between knees as disease activity. All analyses were performed in SAS Enterprise Guide (version 8.3; Cary, NC).

## 3 Results

Fig. 1 illustrates the selection criteria for the 1386 eligible knees (n ​= ​693 participants). The eligible participants were similar to those excluded (Table 1), except the eligible cohort was more likely to have worse knee osteoarthritis severity (frequent knee symptoms and greater Kellgren–Lawrence grade), which reflects the eligibility criteria.

**Table 1:** Descriptive characteristics of participants in the Osteoarthritis Initiative that were eligible or excluded for these analyses.

	Eligible for analyses	Excluded from analyses
	n ​= ​1386 (693 people)	n ​= ​8206 (4103 people)^a^
**Person-level characteristics**		
Age (years)	61.5 (9.1)	62.4 (9.2) [nmiss ​= ​300]
Body mass index (kg/m^2^)	29.6 (4.5) [nmiss ​= ​4]	28.3 (4.9) [nmiss ​= ​549]
Females	398 (57 ​%)	2406 (59 ​%)
White^a^	553 (80 ​%)	3237 (79 ​%)
Black or African American^a^	129 (19 ​%)	745 (18 ​%)
**Knee-level characteristics**		
Kellgren–Lawrence grade		
0	171 (12 ​%)	2942 (36 ​%)
1	288 (21 ​%)	1157 (14 ​%)
2	570 (41 ​%)	1651 (20 ​%)
3	288 (21 ​%)	942 (11 ​%)
4	69 (5 ​%)	286 (3 ​%)
Knee replacement	0 (0 ​%)	69 (1 ​%)
Missing	0 (0 ​%)	1159 (14 ​%)
Frequent knee symptoms	641 (46 ​%)	2010 (24 ​%)

a Other races not reported due to small sample sizes.

Table 2 shows the primary findings that contralateral osteoarthritis severity is associated with disease activity. Specifically, disease activity among knees without radiographic osteoarthritis had a statistically significant relationship with contralateral disease activity (range of odds ratios: 4.86 to 23.22) but not contralateral Kellgren–Lawrence Grade (range of odds ratios: 0.86 to 1.01). Additionally, disease activity among knees with radiographic osteoarthritis had a statistically significant relationship with contralateral disease activity and contralateral Kellgren–Lawrence Grade; however, the association was stronger for contralateral disease activity than contralateral Kellgren–Lawrence Grade (range of odds ratios: 3.67 to 21.29 versus 1.96 to 2.20). When we explored the study knees stratified by Kellgren–Lawrence grade, we consistently observed statistically significant relationships with contralateral disease activity but not contralateral Kellgren–Lawrence grades (Supplemental Table 1). People without a history of injury in both knees had similar associations between knees as those with a history of injury in either knee (Supplemental Table 2). Finally, when we explored each key component of disease activity (effusion-synovitis and BML volumes), we consistently observed statistically significant relationships between knees (Supplemental Tables 3 and 4).

**Table 2:** Disease activity is associated with contralateral osteoarthritis severity.

Strata: Radiographic Severity of Study Knee (Outcome)	Contralateral Knee Status (Exposure)	Tertiles of disease activity in the study knee			Odds ratios (95 ​% CI) Adjusted for gender and age	
		Low (range:−3.3 to −2.0)	Moderate (range:−2.0 to −0.2)	High (range:−0.2 to 34.9)	Moderate vs. Low	High vs. Low
Knees without radiographic OA						
Contralateral KL ​= ​0 or 1		124 (51 ​%)	70 (51 ​%)	40 (52 ​%)	Reference	Reference
Contralateral KL ​= ​2		79 (32 ​%)	44 (32 ​%)	22 (29 ​%)	0.99 (0.61, 1.58)	0.86 (0.47, 1.56)
Contralateral KL ​= ​3 or 4		41 (17 ​%)	24 (17 ​%)	15 (19 ​%)	1.01 (0.56, 1.81)	1.00 (0.49, 2.01)
Contralateral disease activity – Low (−3.3 to −2.0)		154 (63 ​%)	34 (25 ​%)	9 (12 ​%)	Reference	Reference
Contralateral disease activity – Moderate (−2.0 to −0.2)		54 (22 ​%)	66 (48 ​%)	18 (23 ​%)	**5.63 (3.34, 9.51)**	**5.54 (2.33, 13.19)**
Contralateral disease activity – High (−0.2 to 34.9)		36 (15 ​%)	38 (28 ​%)	50 (65 ​%)	**4.86 (2.69, 8.81)**	**23.22 (10.38, 51.93)**
Knees with radiographic OA						
Contralateral KL ​= ​0 or 1		73 (33 ​%)	68 (21 ​%)	84 (22 ​%)	Reference	Reference
Contralateral KL ​= ​2		91 (42 ​%)	162 (50 ​%)	172 (45 ​%)	**2.20 (1.43, 3.38)**	**1.93 (1.27, 2.93)**
Contralateral KL ​= ​3 or 4		54 (25 ​%)	95 (29 ​%)	128 (33 ​%)	**1.96 (1.21, 3.15)**	**2.09 (1.32, 3.30)**
Contralateral disease activity – Low (−3.3 to −2.0)		128 (59 ​%)	84 (26 ​%)	53 (14 ​%)	Reference	Reference
Contralateral disease activity – Moderate (−2.0 to −0.2)		64 (29 ​%)	164 (50 ​%)	97 (25 ​%)	**4.01 (2.68, 6.00)**	**3.67 (2.33, 5.79)**
Contralateral disease activity – High (−0.2 to 34.9)		26 (12 ​%)	77 (24 ​%)	234 (61 ​%)	**4.46 (2.63, 7.56)**	**21.29 (12.64, 35.86)**

Percentages by column.

## 4 Discussion

We found that contralateral knee osteoarthritis severity related to a composite score for the total volume of BMLs and effusion-synovitis. This association was more pronounced when MR imaging assessed disease activity in the contralateral knee instead of relying on radiographic severity, especially among study knees without radiographic osteoarthritis. It is likely beneficial to recognize the contralateral knee is important and not an innocent bystander to achieve optimal outcomes when treating knee osteoarthritis. This work suggests that future investigations should explore if investigators may benefit from using bilateral knee MR imaging for clinical trial eligibility and efficacy assessments, especially in prevention trials. Furthermore, it may be beneficial to explore the need to intervene bilaterally to optimize outcomes when doing localized treatments.

Our current analyses build on prior studies suggesting that disease severity in a knee is related to contralateral radiographic knee osteoarthritis severity. For example, 91 ​% of participants with symptomatic moderate-severe radiographic knee osteoarthritis had an equal or worse radiographic severity grade in the less painful knee [6]. Radiographically normal knees with contralateral radiographic knee osteoarthritis have a higher prevalence of osteoarthritis-related pathology (i.e., Hoffa synovitis, BMLs, and cartilage lesions) than knees with bilateral radiographically normal knees [8]. Furthermore, our study complements findings from a prior clinical trial when the authors suggested that standard weight-bearing posterior–anterior knee radiographs lack sufficient sensitivity to assess the disease severity [15]. We expanded on the existing literature by demonstrating that MR imaging is a valuable tool for understanding the relationship between disease severity in both knees.

While this study offers novel insights into the association of disease severity between knees, it is important to acknowledge its limitations. First, this was a cross-sectional study; however, these findings highlight the need for longitudinal studies to clarify if confounding factors cause these associations and, if not, what mechanism(s) cause a knee to be influenced by the contralateral disease severity. A longitudinal study would also help confirm the negative impact of the contralateral knee and refute the hypothesis that the two knees worsen in parallel. Another limitation is the reliance on a convenience sample selected for a different purpose. However, this study sample created an unprecedented opportunity to study participants with bilateral MR-based measures of osteoarthritis severity. Relying on a convenience sample is unlikely to alter the take-home messages that disease severity in a knee is associated with contralateral knee osteoarthritis severity and that MR imaging may offer a more sensitive assessment of contralateral osteoarthritis severity than standard radiographs.

In conclusion, this study indicated that the structural findings in one knee may relate to structural findings in the other knee. This highlights the need for future studies to explore how the contralateral knee could impact future clinical trial screening, monitoring, and intervention strategies. For example, we may benefit from bilateral knee MR imaging for clinical trial eligibility and efficacy assessments and intervening bilaterally to optimize outcomes when doing localized treatments, especially for prevention trials.

## 5 Author contributions

Jeffrey B. Driban contributed substantially to the 1) conception/design of the work, 2) acquisition, analysis, and interpretation of data for the work, and 3) drafting of the manuscript.

Jonggyu Baek contributed substantially to the 1) conception/design of the work, 2) analysis and interpretation of data for the work, and 3) drafting of the manuscript.

Julieann C. Patarini contributed substantially to the acquisition of data for the work.

Emily Kirillov contributed substantially to the acquisition of data for the work.

Nhung Vo contributed substantially to the acquisition of data for the work.

Michael J. Richard contributed substantially to the acquisition of data for the work.

Ming Zhang contributed substantially to the 1) conception/design of the work and 2) acquisition, analysis, and interpretation of data for the work.

Matthew S. Harkey contributed substantially to the 1) conception/design of the work and 2) acquisition and interpretation of data for the work.

Grace H. Lo contributed substantially to the 1) conception/design of the work and 2) interpretation of data for the work.

Shao-Hsien Liu contributed substantially to the 1) conception/design of the work and 2) interpretation of data for the work.

Charles B Eaton contributed substantially to the 1) conception/design of the work and 2) interpretation of data for the work.

Jamie MacKay contributed substantially to the 1) conception/design of the work, and 2) acquisition and interpretation of data for the work.

Mary F. Barbe contributed substantially to the interpretation of data for the work.

Timothy E. McAlindon contributed substantially to the 1) conception/design of the work, 2) acquisition, analysis, and interpretation of data for the work, and 3) drafting of the manuscript.

All authors contributed to reviewing the work critically for important intellectual content, provided final approval of the version to be published, and agreed to be accountable for all aspects of the work in ensuring that questions related to the accuracy or integrity of any part of the work are appropriately investigated and resolved.

## Funding sources

These analyses were financially supported by a grant from the 10.13039/100000069National Institute of Arthritis and Musculoskeletal and Skin Diseases of the National Institutes of Health under Award Number R01-AR076411. The OAI is a public-private partnership comprised of five contracts (N01-AR-2-2258; N01-AR-2-2259; N01-AR-2-2260; N01-AR-2-2261; N01-AR-2-2262) funded by the 10.13039/100000002National Institutes of Health, a branch of the Department of Health and Human Services, and conducted by the OAI Study Investigators. Private funding partners include Merck Research Laboratories; Novartis Pharmaceuticals Corporation; GlaxoSmithKline; and Pfizer, Inc. Private sector funding for the OAI is managed by the Foundation for the National Institutes of Health. This manuscript was prepared using an OAI public use data set and does not necessarily reflect the opinions or views of the OAI investigators, the NIH, or the private funding partners. This work was supported in part with resources at the VA's Health Services Research and Development Service Center for Innovations in Quality, Effectiveness, and Safety (#CIN 13–413) at the Michael E. DeBakey VA Medical Center, Houston, TX. The views expressed in this article are those of the authors and do not necessarily represent the views of the Department of Veterans Affairs. The funding sources had no role in study design; in the collection, analysis, and interpretation of data; in the writing of the report; nor in the decision to submit the article for publication.

## Conflict of interest

Jeffrey Driban reports financial support was provided by National Institute of Arthritis and Musculoskeletal and Skin Diseases. Grace Lo reports financial support was provided by VA Health Services Research and Development Service Center for Innovations in Quality, Effectiveness, and Safety. Timothy McAlindon reports a relationship with Sanofi that includes: consulting or advisory. Timothy McAlindon reports a relationship with Kolon TissueGene that includes: consulting or advisory. Timothy McAlindon reports a relationship with Medidata that includes: consulting or advisory. Timothy McAlindon reports a relationship with Organogenesis Inc that includes: consulting or advisory. Timothy McAlindon reports a relationship with Ambulomics that includes: equity or stocks. Timothy McAlindon reports a relationship with Arthrometrics that includes: equity or stocks. Matthew Harkey reports a relationship with Osteoarthritis Research Society International that includes: board membership. Jeffrey Driban, Timothy McAlindon, Ming Zhang have patent #Objective Assessment of Joint Damage (US-20220202356) pending to Tufts Medical Center, Inc. All other authors declare that they have no known competing financial interests or personal relationships that could have appeared to influence the work reported in this paper.
