# Supplementary material for: Contralateral knee osteoarthritis severity relates to magnetic resonance imaging findings in knees with and without osteoarthritis: Data from the osteoarthritis initiative
Source: Osteoarthr Cartil Open. 2025 Feb 17;7(2):100585. doi: 10.1016/j.ocarto.2025.100585 (PMC11908568; doi:10.1016/j.ocarto.2025.100585)
Supplement: Multimedia component 1 [file mmc1.docx]

| **Supplemental Table 1. Disease Activity is Associated with Contralateral Disease Activity but Rarely Radiographic Severity** | | | | | | |
| --- | --- | --- | --- | --- | --- | --- |
|  |  | Tertiles of Disease Activity  in the Study Knee | | | Unadjusted Odds Ratios (95% CI) | |
| Strata: Radiographic Severity of Study Knee | Contralateral Knee Status | Low  (range:  -3.3 to -2.0) | Moderate  (range:  -2.0 to -0.2) | High  (range:  -0.2 to 34.9) | Moderate vs. Low | High vs. Low |
| Study Knees KL = 0 |  |  |  |  |  |  |
|  | Contralateral KL = 0 or 1 | 36 (38%) | 12 (26%) | 11 (38%) | Reference | Reference |
|  | Contralateral KL = 2 | 36 (38%) | 22 (50%) | 11 (38%) | 1.90 (0.82, 4.39) | 0.97 (0.37, 2.55) |
|  | Contralateral KL = 3 or 4 | 24 (25%) | 11 (24%) | 7 (24%) | 1.38 (0.52, 3.63) | 0.96 (0.32, 2.86) |
|  |  |  |  |  |  |  |
| Contralateral Disease Activity – Low (-3.3 to -2.0) | | 49 (51%) | 10 (22%) | 3 (10%) | Reference | Reference |
| Contralateral Disease Activity – Moderate (-2.0 to -0.2) | | 28 (29%) | 19 (41%) | 9 (31%) | **3.43 (1.39, 8.45)** | **5.70 (1.40, 23.23)** |
| Contralateral Disease Activity – High (-0.2 to 34.9) | | 19 (20%) | 17 (37%) | 17 (57%) | **4.59 (1.77, 11.90)** | **16.53 (4.22, 64.66)** |
| Study Knees KL = 1 |  |  |  |  |  |  |
|  | Contralateral KL = 0 or 1 | 88 (59%) | 58 (63%) | 29 (60%) | Reference | Reference |
|  | Contralateral KL = 2 | 43 (29%) | 21 (23%) | 11 (23%) | 0.74 (0.40, 1.38) | 0.78 (0.36, 1.72) |
|  | Contralateral KL = 3 or 4 | 17 (11%) | 13 (14%) | 8 (17%) | 1.16 (0.52, 2.57) | 1.41 (0.55, 3.62) |
|  |  |  |  |  |  |  |
| Contralateral Disease Activity – Low (-3.3 to -2.0) | | 105 (71%) | 24 (26%) | 6 (13%) | Reference | Reference |
| Contralateral Disease Activity – Moderate (-2.0 to -0.2) | | 26 (18%) | 47 (51%) | 9 (19%) | **7.96 (4.14, 15.32)** | **6.33 (2.05, 19.53)** |
| Contralateral Disease Activity – High (-0.2 to 34.9) | | 17 (11%) | 21 (23%) | 33 (69%) | **5.47 (2.51, 11.95)** | **36.93 (13.22, 103.16)** |
| Study Knees KL = 2 |  |  |  |  |  |  |
|  | Contralateral KL = 0 or 1 | 64 (37%) | 50 (23%) | 31 (18%) | Reference | Reference |
|  | Contralateral KL = 2 | 76 (44%) | 130 (59%) | 104 (59%) | **2.19 (1.37, 3.49)** | **2.83 (1.68, 4.76)** |
|  | Contralateral KL = 3 or 4 | 32 (19%) | 41 (19%) | 42 (24%) | 1.64 (0.91, 2.96) | **2.71 (1.45, 5.08)** |
|  |  |  |  |  |  |  |
| Contralateral Disease Activity – Low (-3.3 to -2.0) | | 103 (60%) | 51 (23%) | 16 (9%) | Reference | Reference |
| Contralateral Disease Activity – Moderate (-2.0 to -0.2) | | 49 (28%) | 111 (50%) | 46 (26%) | **4.71 (2.92, 7.60)** | **6.10 (3.14, 11.84)** |
| Contralateral Disease Activity – High (-0.2 to 34.9) | | 20 (12%) | 59 (27%) | 115 (65%) | **6.07 (3.30, 11.18)** | **37.22 (18.30, 75.69)** |
| Study Knees KL = 3 or 4 |  |  |  |  |  |  |
|  | Contralateral KL = 0 or 1 | 9 (20%) | 18 (17%) | 53 (26%) | Reference | Reference |
|  | Contralateral KL = 2 | 15 (33%) | 32 (31%) | 68 (33%) | 1.05 (0.38, 2.88) | 0.76 (0.31, 1.88) |
|  | Contralateral KL = 3 or 4 | 22 (48%) | 54 (52%) | 86 (42%) | 1.21 (0.47, 3.10) | 0.66 (0.28, 1.54) |
|  |  |  |  |  |  |  |
| Contralateral Disease Activity – Low (-3.3 to -2.0) | | 25 (54%) | 33 (32%) | 37 (18%) | Reference | Reference |
| Contralateral Disease Activity – Moderate (-2.0 to -0.2) | | 15 (33%) | 53 (51%) | 51 (25%) | **2.71 (1.25, 5.90)** | **2.30 (1.07, 4.95)** |
| Contralateral Disease Activity – High (-0.2 to 34.9) | | 6 (13%) | 18 (17%) | 119 (57%) | **2.18 (0.75, 6.32)** | **13.44 (5.11, 35.36)** |
| Percentages by column | | | | | | |

| **Supplemental Table 2. Disease Activity is Associated with Contralateral Osteoarthritis Severity** **– Stratified by History of Injury in Either Knee** | | | | | | | |
| --- | --- | --- | --- | --- | --- | --- | --- |
|  |  | Tertiles of Disease Activity  in the Study Knee | | | Odds Ratios (95% CI)  Adjusted for gender and age | | |
| Strata: History of Injury | Contralateral Knee Status  (Exposure) | Low  (range:  -3.3 to -2.0) | Moderate  (range:  -2.0 to -0.2) | High  (range:  -0.2 to 34.9) | Moderate vs. Low | High vs. Low | |
| No History of Injury in Both Knees (n = 638 knees) | | | | | | | |
|  | Contralateral KL = 0 or 1 | 88 (44%) | 65 (30%) | 66 (30%) | Reference | Reference | |
|  | Contralateral KL = 2 | 74 (37%) | 103 (48%) | 93 (42%) | **1.93 (1.23, 3.01)** | **1.73 (1.10, 2.71)** | |
|  | Contralateral KL = 3 or 4 | 40 (20%) | 48 (22%) | 61 (28%) | 1.62 (0.95, 2.77) | **2.04 (1.21, 3.43)** | |
|  |  |  |  |  |  |  | |
| Contralateral Disease Activity – Low (-3.3 to -2.0) | | 122 (60%) | 54 (25%) | 26 (12%) | Reference | Reference | |
| Contralateral Disease Activity – Moderate (-2.0 to -0.2) | | 54 (27%) | 108 (50%) | 54 (25%) | **4.60 (2.91, 7.29)** | **4.60 (2.61, 8.12)** | |
| Contralateral Disease Activity – High (-0.2 to 34.9) | | 26 (13%) | 54 (25%) | 140 (64%) | **4.61 (2.61, 8.14)** | **25.51 (14.03, 46.38)** | |
| History of Injury in Either Knee (n = 732 knees) | | | | | | | |
|  | Contralateral KL = 0 or 1 | 105 (42%) | 68 (28%) | 57 (24%) | Reference | Reference | |
|  | Contralateral KL = 2 | 93 (37%) | 102 (42%) | 99 (42%) | **1.81 (1.19, 2.76)** | **2.09 (1.35, 3.25)** | |
|  | Contralateral KL = 3 or 4 | 55 (22%) | 71 (29%) | 82 (34%) | **1.92 (1.20, 3.08)** | **2.53 (1.57, 4.08)** | |
|  |  |  |  |  |  |  | |
| Contralateral Disease Activity – Low (-3.3 to -2.0) | | 154 (61%) | 63 (26%) | 36 (15%) | Reference | Reference | |
| Contralateral Disease Activity – Moderate (-2.0 to -0.2) | | 63 (25%) | 118 (49%) | 60 (25%) | **4.43 (2.89, 6.77)** | **3.85 (2.31, 6.42)** | |
| Contralateral Disease Activity – High (-0.2 to 34.9) | | 36 (14%) | 60 (25%) | 142 (60%) | **3.85 (2.31, 6.41)** | **15.32 (9.12, 25.76)** | |
| Percentages by column  16 knees could not be classified based on a history of injury because of missing data. | | | | | | |  |

| **Supplemental 3. Effusion-Synovitis Volume is Associated with Contralateral Osteoarthritis Severity** | | | | | | | |
| --- | --- | --- | --- | --- | --- | --- | --- |
|  |  | Tertiles of Effusion-Synovitis  in the Study Knee | | | Odds Ratios (95% CI)  Adjusted for gender and age | | |
| Strata: Radiographic Severity of Study Knee (Outcome) | Contralateral Knee Status  (Exposure) | Low  (range:  1.9 to 8.2) | Moderate  (range:  8.2 to 13.3) | High  (range:  13.3 to 81.4) | Moderate vs. Low | High vs. Low | |
| Knees without Radiographic OA | | | | | | | |
| Contralateral Effusion-Synovitis Volume – Low (1.9 to 8.2 cm^3^) | | 123 (57%) | 46 (26%) | 7 (10%) | Reference | Reference | |
| Contralateral Effusion-Synovitis Volume – Moderate (8.2 to 13.3 cm^3^) | | 67 (31%) | 63 (36%) | 30 (44%) | **2.33 (1.42, 3.81)** | **6.68 (2.76, 16.21)** | |
| Contralateral Effusion-Synovitis Volume – High (13.3 to 81.4 cm^3^) | | 26 (12%) | 66 (38%) | 31 (46%) | **6.11 (3.42, 10.91)** | **16.14 (6.31, 41.31)** | |
| Knees with Radiographic OA | | | | | | | |
| Contralateral Effusion-Synovitis Volume – Low (-3.3 to -2.0) | | 155 (63%) | 81 (28%) | 50 (13%) | Reference | Reference | |
| Contralateral Effusion-Synovitis Volume – Moderate (-2.0 to -0.2) | | 60 (24%) | 117 (40%) | 127 (32%) | **3.45 (2.28, 5.23)** | **6.04 (3.86, 9.44)** | |
| Contralateral Effusion-Synovitis Volume – High (-0.2 to 34.9) | | 31 (13%) | 91 (31%) | 217 (55%) | **5.26 (3.21, 8.61)** | **20.25 (12.31, 33.29)** | |
| Percentages by column | | | | | | |  |

| **Supplemental 4. Bone Marrow Lesion Volume is Associated with Contralateral Osteoarthritis Severity** | | | | | | | |
| --- | --- | --- | --- | --- | --- | --- | --- |
|  |  | Tertiles of Bone Marrow Lesion  in the Study Knee | | | Odds Ratios (95% CI)  Adjusted for gender and age | | |
| Strata: Radiographic Severity of Study Knee (Outcome) | Contralateral Knee Status  (Exposure) | Low  (range:  0.0 to 1.0) | Moderate  (range:  1.0 to 4.0) | High  (range:  4.0 to 97.3) | Moderate vs. Low | High vs. Low | |
| Knees without Radiographic OA | | | | | | | |
| Contralateral BML Volume – Low (0.0 to 1.0 cm^3^) | | 141 (58%) | 45 (29%) | 12 (19%) | Reference | Reference | |
| Contralateral BML Volume – Moderate (1.0 to 4.0 cm^3^) | | 64 (26%) | 72 (47%) | 15 (24%) | **3.49 (2.17, 5.62)** | **2.83 (1.25, 6.42)** | |
| Contralateral BML Volume – High (4.0 to 97.3 cm^3^) | | 38 (16%) | 35 (24%) | 36 (57%) | **2.91 (1.65, 5.14)** | **11.62 (5.48, 24.65)** | |
| Knees with Radiographic OA | | | | | | | |
| Contralateral BML Volume – Low (0.0 to 1.0 cm^3^) | | 131 (60%) | 75 (24%) | 59 (15%) | Reference | Reference | |
| Contralateral BML Volume – Moderate (1.0 to 4.0 cm^3^) | | 56 (25%) | 142 (46%) | 113 (28%) | **4.42 (2.90, 6.73)** | **4.50 (2.88, 7.03)** | |
| Contralateral BML Volume – High (4.0 to 97.3 cm^3^) | | 33 (15%) | 92 (30%) | 226 (57%) | **4.85 (2.97, 7.91)** | **15.22 (9.41, 24.61)** | |
| Percentages by column | | | | | | |  |
